# Synergistic effect of κ-carrageenan on oxazolone-induced inflammation in BALB/c mice

**DOI:** 10.1186/s12876-016-0459-7

**Published:** 2016-03-25

**Authors:** Wei Wu, Feng Wang, Xin Gao, Tingting Niu, Xiaojuan Zhu, Xiaojun Yan, Haimin Chen

**Affiliations:** Key Laboratory of Applied Marine Biotechnology of Zhejiang Province, Ningbo University, Post Box 71, Ningbo, Zhejiang 315211 China; Department of Clinical Laboratory, Lihuili hospital of Ningbo Medical Center, Ningbo, Zhejiang 315041 China; Collaborative Innovation Center for Zhejiang Marine High-efficiency and Healthy Aquaculture, Ningbo University, Ningbo, Zhejiang 315211 China

**Keywords:** κ-carrageenan, Oxazolone, Intestinal inflammation, Aggravation

## Abstract

**Background:**

Carrageenan is a traditional ingredient that has been widely used in the food industry. In the present study, we propose a hypothesis that carrageenan is a conditional inflammatory agent. When the intestinal tract is in an “unhealthy” state such as that during bacterial infection or acute inflammation, carrageenan can synergistically enhance the inflammatory response.

**Methods:**

BALB/C mice received κ-carrageenan via intragastric administration prior to the induction of oxazolone colitis. Weight changes, survival rate, histologic change, secretion of inflammatory cytokines, ratio of regulatory T cells (Tregs) in peripheral blood, and expression of genes and proteins involved in inflammation and cell proliferation in the colonic mucosa were examined.

**Results:**

Intragastric administration of κ-carrageenan to BALB/c mice prior to the induction of oxazolone colitis resulted in an aggravation of body weight loss, a decrease in the survival ratio, aggravation of colonic inflammation, and decrease in the ratio of CD4 + CD25+/CD4+. The secretion of interleukin-4 (IL-4), interleukin-10 (IL-10), tumor necrosis factor-α (TNF-α), and interleukin-6 (IL-6) also significantly increased after κ-carrageenan administration. κ-Carrageenan, together with oxazolone, suppressed the expression of forkhead box p3 (FOXp3) and increased the expression of Toll-like receptor 4 (TLR4), Nuclear factor-κB (NF-κB), and proliferating cell nuclear antigen in the colonic mucosa. These results were confirmed by qRT-PCR and western blot analyses at the molecular and protein levels, respectively.

**Conclusions:**

κ-Carrageenan aggravated oxazolone-induced intestinal inflammation in BALB/c mice. This effect is associated with an activation of the TLR4-NF-κB pathway, a decreased ratio of Tregs, and the induction of Th2-dependent immune responses.

## Background

Carrageenan, a high-molecular weight saccharide, is used widely as a thickener, stabilizer, emulsifier, and texturizer in a variety of processed foods (including infant formula, whipped cream, cottage cheese, ice cream, and nutritional supplements) and other products (i.e., pharmaceuticals, cosmetics, and toothpaste) [1–3]. Several international agencies have earlier reported that carrageenan is safe for human consumption [4, 5]. However, investigators later demonstrated that carrageenan induces gastrointestinal ulcerations and cancer in animal models [2, 4]. Carrageenan has been widely used in experimental models of inflammation to assess the activity of anti-inflammatory drugs and to study mediators of inflammation [6]. Therefore, the concept of safety of carrageenan has remained highly controversial.

Current research studies on carrageenan have mainly focused on its effects at the organismal and cellular levels. Animal models such as rats [6], guinea pigs [7], pig [8], mouse [9], and rhesus monkeys [10] have demonstrated that carrageenan induces colorectal tumors and ulcerative colitis. However, several international agencies have concluded that carrageenan is harmless [4, 5]. The US Food and Drug Administration (FDA) has declared food-grade carrageenan as generally recognized as safe (GRAS) for use as a food ingredient [11]. Tobacman et al. conducted extensive research studies to evaluate the safety of carrageenan at the cellular level [2, 12–19]. They observed that exposure of human intestinal epithelial cells to food-grade carrageenan triggered a distinct inflammatory response via the activation of Bcl10 in the NF-κB pathway [20], as well as up regulation of interleukin-8 (IL-8) secretion [16, 20], and identified that the Toll-like receptor 4 (TLR4) is the surface membrane receptor for carrageenan in human colonic epithelial cells. Bernard et al. observed that carrageenan induces tumor necrosis factor alpha (TNF-α) production in human monocytes [21]. Although an upregulation of inflammatory cytokines in human cells occurs following food-grade carrageenan exposure, we found that the increase was actually low, only 2–3-fold higher than that observed in the blank control [16, 20]. Is this difference in expression level enough to cause a strong inflammatory reaction in vivo? Although lipopolysaccharides, which are highly proinflammatory molecules, can increase the secretion of IL-8 and TNF-α in macrophages by a dozen- to a hundred-fold [22], the inflammatory mechanism of carrageenan remains elusive.

In our previous studies, we observed that λ-carrageenan enhances the LPS-induced production of IL-8 at the cellular level [23]. Moreover, when differentiated human colonic epithelial cells were co-cultured with macrophage cells, the epithelial monolayers were significantly and easily damaged by carrageenan. Based on these earlier findings, we hereby propose the hypothesis that carrageenan is a conditional inflammatory agent. When the intestinal tract is in an “unhealthy” state such as that during bacterial infection or acute inflammation, carrageenan can synergistically enhance the inflammatory response.

Therefore, in the present study, an oxazolone (OXA)-induced mild intestinal inflammation model was developed to investigate whether κ-carrageenan can enhance the inflammatory responses of the gut with acute inflammation. Furthermore, the mechanism underlying this inflammatory response was also investigated in relation to the TLR4 pathway.

## Methods

### Animals

Seven-week-old male and female BALB/c mice with weights ranging from 20-25 g were purchased (SCXK 2012–0001, Beijing Weitong Lihua Experimental Animal Technology Co., Ltd.), housed in the animal center of Ningbo University Medicine College (Ningbo, China), and used in the present study. Five same-sex mice were housed in a cage and maintained under a 12-h light/12-h dark cycle (08:00 AM lights on) with food and water provided ad libitum. Housing and experimental environments were temperature- and humidity-controlled (21 ± 2 °C and approximately 60 %, respectively). All mice were allowed to habituate to the housing environment for 3 days prior to gavage administration. All experimental procedures were performed in accordance with the National Institutes of Health Guide for the Care and Use of Laboratory Animals, and approved by the Ethical Committee of Animal Use and Protection of Ningbo University Health Science Center.

### Animal experimental procedure

Eighty mice were divided into eight groups and placed in clear plastic cages with a mesh top under specific pathogen-free conditions, with each experimental group consisting of four males and four females. The experimental groups were designed as follows: the blank group only received a saline solution; the OXA group was treated with 150 μL of 1 % OXA; the carrageenan groups were treated with different concentrations of carrageenan, namely, the low-dose group (1.7 mg/kg, LOW), medium-dose group (8.3 mg/kg, MED), and high-dose group (41.7 mg/kg, HIG); carrageenan + OXA groups received low, medial, and high doses of carrageenan following by the administration of OXA and marked as “LOW + OXA,” “MED + OXA,” and “HIG+ OXA.”

κ-Carrageenan (Sigma Chemical Co., St Louis, MO, USA) was dissolved in saline with vigorous stirring at 37 °C and administered at three different doses, 1.7 mg/kg, 8.3 mg/kg, and 41.7 mg/kg by gavage administration. These doses were decided according to the data of preliminary experiments based on related study [18]. To assess the synergistic effect of carrageenan on OXA-induced inflammation, different doses of carrageenan were gavage administered for two weeks prior to OXA administration.

OXA-induced intestinal inflammation was established as previously described by Heller et al., with minor modifications [24]. To presensitize mice, a 2 × 2 cm area of the abdominal skin was shaved, and 200 μL of OXA [a 3 % (w/v) solution in 100 % ethanol, Sigma-Aldrich, St. Louis, MO] was smeared 5 days before OXA was administered. Five days after presensitization (day 0), the mice were rechallenged intrarectally with 100 μL of 1 % OXA in 50 % ethanol or only 50 % ethanol (vehicle, Blank group) under general anesthesia with isoflurane (Baxter, Deerfield, IL, USA). Intrarectal injection was administered with a polyurethane umbilical catheter. To ensure distribution of the OXA throughout the entire colon, the mice were held head down in a vertical position for 60 s after injection.

The mice were then monitored for symptoms of diarrhea, loss of body weight, and death the next day (day 1). The mice underwent exsanguination by cardiac puncture and the collected blood was used to determine the CD4^+^CD25^+^ Treg ratio and cytokine levels by flow cytometry. The colon was also isolated; one portion was used in macroscopic evaluation, whereas the rest was fixed in paraformaldehyde (Merck, Darmstadt, Germany) or glutaraldehyde solution for immunohistochemical and scanning electron microscopy analysis.

### Macroscopic evaluation

Macroscopic evaluation of colonic tissues used the following criteria: 0, normal; 1, erythema only; 2, erythema, slight edema, and small erosions; 3, two or more bleeding ulcers and/or inflammation and/or moderate adhesions; and 4, severe ulceration and/or stenosis with dilations and/or severe adhesions.

### Histological analysis

The colonic tissues were dissected and washed with Hank's balanced salt solution (containing 10 μg/mL of gentamicin, 100 U/mL of penicillin, and 100 μg/mL of streptomycin). The tissues were then fixed in 10 % natural buffered formalin, embedded in paraffin, cut into tissue sections (5-μm-thick), and stained with hematoxylin and eosin (H&E).

Histological examination was evaluated as previously described [25]. The degree of histologic damage and inflammation was graded in a blinded fashion by expert histologists. The following manifestations were included in the evaluation: the amount of inflammation (0, none; 1, mild; 2, moderate; 3, severe; and 4, accumulation of inflammatory cells in the gut lumen), distribution of lesions (0, none; 1, focal; 2, multifocal; 3, nearly diffuse; and 4, diffuse), depth of inflammation and layers involved (0, none; 1, mucosa only; 2, mucosa and submucosa; 3, limited transmural involvement; and 4, transmural), and nature of mucosal changes (0, none; 1, minimal degeneration; 2, more degeneration; and 3, more necrosis). The overall histologic score was the sum of the four manifestations (maximum score: 15).

### Intestinal mucosal morphology

The processing of intestinal segments for scanning electron microscopy was conducted as described elsewhere [26]. Briefly, one segment of the tissue sample was fixed with 2.5 % glutaraldehyde. The segments were washed twice in phosphate buffer (pH = 7.2) and then post-fixed in osmium tetroxide (1 % in phosphate buffer, pH = 7.2, 2 h). After a series of dehydration steps in alcohol from 30 % to 100 %, the segments were critical point-dried (CPD030; BAL-TEC; Balzers; Liechtenstein), mounted on Al stubs, sputter-coated with gold by using a high-resolution fine coater (SCD005; BAL-TEC; Balzers; Liechtenstein), and then examined under a Jeol JSM-6301 scanning microscope (JEOL Ltd., Musashino, Tokyo, Japan). Morphological evaluation of the intestinal mucosal and microvilli was then performed. Scanning electron microscopic images (×200 magnification) were analyzed to measure the histological changes on the epithelial cell surface.

### Determination of CD4 + CD25 + Treg ratio by flow cytometry

A total of 2.5 μL of anti-mouse CD3-eFluor450, 0.625 μL of anti-mouse CD4-PE, 0.625 μL of anti-mouse CD25-APC (all from eBioscience, California, USA) were added to 100 μL of peripheral blood. Following incubation at room temperature in the dark for 15–30 min, 1 mL of erythrocyte lysis solution was added to the samples and incubated for 15–20 min. The cells were washed with 2 mL phosphate-buffered saline (PBS) and resuspended in 500 μL PBS. The cells were then analyzed using a Beckman Gallios flow cytometer (Beckman Coulter, Inc., California, USA).

### Immunohistochemical analyses

For immunocytochemical analysis, the mouse colonic tissues were fixed with 4 % paraformaldehyde in PBS, embedded in paraffin, and cut into tissue sections (5-μm-thick). The tissues were blocked with 1 % bovine serum albumin (BSA, Nacalai tesque) for 60 min at room temperature. The primary antibodies used in the present study included anti-TLR4 antibody (1:200, ab22048, Abcam, Cambridge, UK), anti-NF-κB antibody (1:200, 8242, CST, Danvers, MA, USA), anti-FOXp3 antibody (1:200, ab54501, Abcam, Cambridge, UK), and anti-PCNA antibody (1:100, Santa Cruz Biotechnology, Dallas, TX, USA). Secondary antibodies included goat anti-rabbit IgG (PV-9001, ZSGB-BIO, Peking, China) and rabbit anti-mouse IgG (PV-9005, ZSGB-BIO, Peking, China). Negative controls substituted with non-immunized mouse IgG at the same concentrations as that for each primary antibody were employed. Images were captured using a Leitz Dialux 22 Microscope (Leica Microsystems, Wetzlar, Germany) equipped with a QIcam Fast 1394 camera (QImaging, Surrey, BC, Canada). The images were analyzed using the software, Image Pro Plus 6.0 (Media Cybernetics UK, Marlow, UK). The levels of expression of TLR4, NF-κB, FOXp3, and PCNA were determined by measuring the positively labeled areas in relation to the total area.

### Analysis of cytokine levels

Serum samples were collected after centrifugation of blood and stored at −80 °C. The levels of proinflammatory cytokines, including interleukin IL-6, tumor necrosis factor (TNF), Th1 (IL-2, interferon IFN-γ), Th2 (IL-4, IL-10), and Th17 (IL-17) cytokines in each serum sample were determined by using the BD Cytometric Bead Array mouse Th1/Th2/Th17 7 cytokine kit (BD BioSciences, NJ, USA) according to the manufacturer’s instructions. Samples and standards were analyzed on a FACS Calibur flow cytometer (BD BioSciences, NJ, USA). The concentrations were assessed by using FCAP Array software.

### RNA extraction and real-time quantitative reverse transcription-PCR (RT-qPCR) analysis

Total RNA was isolated from scraped colonic mucosa of experimental mice using the TaKaRa RNAiso Plus Reagent (TaKaRa, Dalian, China) following the manufacturer’s instructions and then treated with RNase-free DNase I. Then, 2 μg of total RNA was re-transcribed into cDNA using a total reaction volume of 40 μL following standard M-MLV reverse transcriptase protocols (TaKaRa, Dalian, China). The primers of mouse target genes, including *TLR4*, *NF-κB*, *FOXp3*, and *PCNA*, as well as reference genes *β-actin*, *GAPDH*, and *18S-r*RNA are listed in Table 1. The corresponding PCR products were sequenced by an ABI 3730 automated sequencer (Invitrogen, Carlsbad, CA, USA). To assess PCR efficiency, 10-fold serial dilutions of *TLR4*, *NF-κB*, *FOXp3*, *PCNA*, *β-actin*, *Gapdh*, and *18S*-rRNA plasmid cDNA were used to generate a standard curve for each assay plate. The PCR reaction system included 1 μL of cDNA, 0.4 μM of forward and reverse primers, 10 μL of SYBR Premic Ex Taq II, 7.4 μL of dH_2_O, as recommended by the manufacturer of SYBR-Green I (TaKaRa, Dalian, China). The cycling conditions were 95 °C for 5 min, followed by 40 cycles of 95 °C for 30 s, 60 °C for 1 min, and 72 °C for 15 s, which were conducted on a Mastercycler ep realplex real-time PCR system (Eppendorf, Hamburg, Germany). By using a standard curve, PCR efficiency was calculated (Table 1). After the amplification, melting curves were obtained by slow heating from 60 °C to 95 °C at increments of 0.5 °C/s and continuous fluorescence collection, which confirmed that only our specific product peaks were detected. RT-qPCR analysis of the samples was conducted as earlier described. Relative gene expression was analyzed by using the comparative cycle (Ct) value, which was compared using the relative quantification method. The mRNA expression of *TLR4*, *NF-κB*, *FOXp3*, *PCNA*, *GAPDH*, and *18S*-rRNA were normalized against the expression of *β-actin*.

**Table 1:** Oligonucleotide primers used in this work

Primers	Nucleotide sequence	Primer efficiency	Positive control	Negative control
TLR4-F	GCTGCAACTGATGTTCCTTCT	95 %	N/A	N/A
TLR4-R	CCCAACATTCATCCATCTCA			
NF-κB-F	AAAGCCCTGACAGTCCATTG	93 %	N/A	N/A
NF-κB-R	TTGCTAGACACCGTCTGTGC			
FOXp3-F	TCGAGCTTCCCAGAGAGAGA	94 %	N/A	N/A
FOXp3-R	GGCCCTGACTGGATGTAAGT			
PCNA-F	AAGAAGAGGAGGCGGTAA	92 %	N/A	N/A
PCNA-R	AGTGTCCCATGTCAGCAA			
β-actin-F	TTGCTGACAGGATGCAGAAG	94 %	N/A	N/A
β-actin-R	ACATCTGCTGGAAGGTGGAC			
GAPDH-F	AATGTGTCCGTCGTGGATCT	93 %	N/A	N/A
GAPDH-R	GGTCCTCAGTGTAGCCCAAG			
18S-rRNA-F	ATTGGAGCTGGAATTACCGC	95 %	N/A	N/A
18S-rRNA-R	CGGCTACCACATCCAAGGAA			

Notes: N/A indicates no results be detected

### Preparation of nuclear extracts

To prepare a whole lysate solution, the collected tissues were washed twice with ice-cold PBS and lysed in a lysis buffer (Beyotime, Shanghai, China) that contained 1 mM of phenylmethylsulfonyl fluoride (PMSF) and a protein phosphatase inhibitor cocktail (Roche, Basel, Switzerland) for 10 min on ice. The homogenates were centrifuged at 12,000 rpm at 4 °C for 10 min. Total protein was extracted from the scraped colonic mucosa of the experimental mice using a NE-PER nuclear and Cytoplasmic Extraction kit (Pierce, Rockford, IL, USA), following the manufacturer's recommendations. Protein concentration was determined using a Bio-Rad DC Protein assay reagent (Bio-Rad Laboratories, Inc., Hercules, CA, USA), following the manufacturer's instructions.

### Western blot analysis

Proteins (30 μg) in the nuclear extracts or in the whole lysate were separated by 10 % sodium dodecyl sulfate-polyacrylamide gel (SDS-PAGE), transferred onto polyvinylidene fluoride (PVDF) membranes, and probed with antibodies against TLR4, NF-κB, and p65 antibodies (Santa Cruz Biotechnology, Santa Cruz, CA, USA), followed by appropriate horseradish peroxidase (HRP)-linked secondary antibodies (CST). The immunoreactive proteins were detected by using WesternBright ECL (Advansta Inc., Menlo Park, CA, USA). The results were quantified by measuring the band intensity relative to that of β-actin using the AlphaView™ Software (Alpha Innotech, San Leandro, CA, USA), and expressing as the relative intensities.

### Statistical analysis

Statistical analyses were performed using the SPSS software, version 16.0 (SPSS Inc., Chicago, IL, USA). The results were expressed as means ± SEM. *P* < 0.05 was considered to indicate statistically significant differences. Macroscopic and histological score analyses were performed by using the student’s *t*-test. Differences in weight loss, cytokine levels, ratio of Treg (CD4^+^CD25^+^CD127dim), mRNA expression, western blot data, and average integral optical density of expression of various inflammatory proteins in mouse colonic mucosa among different groups were analyzed by using one-way ANOVA after post hoc testing.

## Results

### κ-Carrageenan aggravates weight loss and decrease in the survival ratio of mice with OXA-induced inflammation

**Fig. 1:**
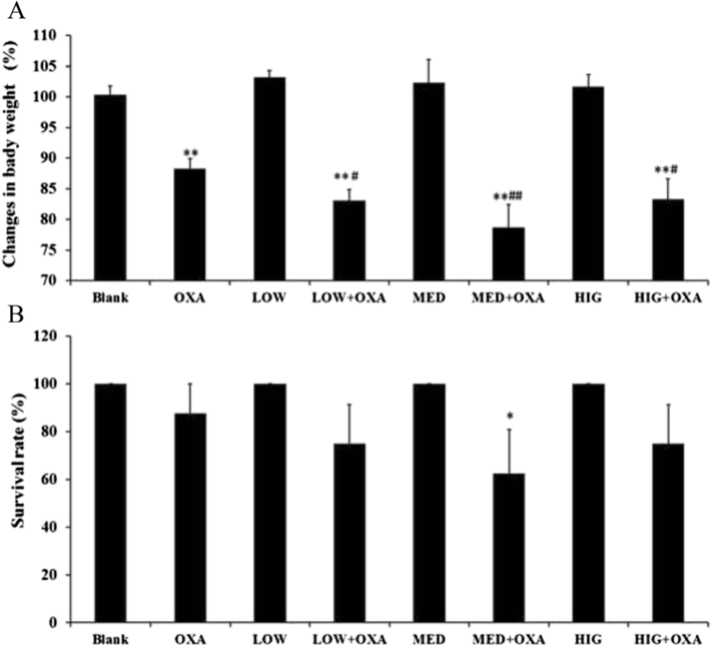
The effect of κ-carrageenan on body weight and survival ratio of mice with oxazolone-induced colitis. **a** Weight changes. **b** Survival ratio. Mice were treated with different doses of carrageenan, and then intrarectally challenged with 100 μL of 1 % oxazolone. One day after treatment, the decrease in body weight and the mortality rate of the animals were recorded. Pairwise comparisons between groups at various time points were conducted by using two-way ANOVA and the LSD post hoc test. ^*^ *P* < 0.05, ^**^ *P* < 0.01, compared with that of the Blank group. ^#^ *P* < 0.05, ^##^ *P* < 0.01, compared to that of the oxazolone-treated group

To determine whether pretreatment with κ-carrageenan affected the damage induced by OXA, weight loss and survival rates of the mouse models were examined. Figure 1a reveals that the percent of weight loss on day 1 was significantly different [F (7, 55) = 30.098, *P* < 0.01]. No significant differences in weight loss between the Blank group and the carrageenan alone-treated groups on day 1 were detected. The rate of weight loss in mice belonging to the OXA-treated group was significantly lower than that observed in Blank group (*P* < 0.01), which decreased by about 12 %. Interestingly, pretreatment with κ-carrageenan aggravated the weight loss. The body weights of mice from the LOW + OXA, MED + OXA, and HIG + OXA groups were markedly lower than that of the OXA-treated group on day 1 (*P* = 0.048, *P* = 0.001, and *P* = 0.047), which decreased by about 16.9 %, 21.3 %, and 16.5 % respectively.

Consistent with the observed difference in weight change, the Blank and carrageenan-treated groups showed similar survival rates, whereas the OXA-treated mice showed an 87.5 % survival ratio. The survival ratio of the carrageenan + OXA group was lower than that of the OXA-treated groups. Among these, the group that received a medial-dose (8.3 mg/kg) κ-carrageenan for 14 days showed a 37.5 % mortality rate, which was the highest (Fig. 1b), suggesting that medial-dose (8.3 mg/kg) κ-carrageenan significantly aggravated weight loss and decreased the survival ratio due to OXA exposure.

### Effects of κ-carrageenan on the severity of colonic damage

**Fig. 2:**
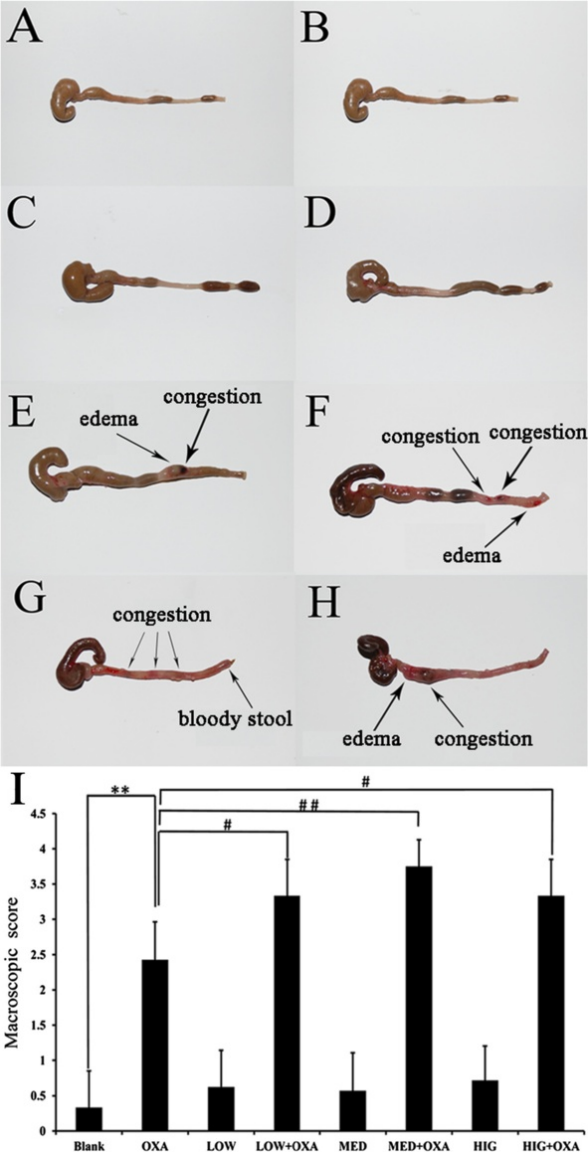
Effect of κ-carrageenan on colonic damage following oxazolone administration. A representative photograph of the colon encompassing the cecum to the rectum. **a** Blank group; **b** LOW group; **c** MED group; **d** HIG group; **e** OXA group; **f** LOW + OXA group; **g** MED + OXA group; **h** HIG + OXA group; **i** Colonic damage scores 1 day after oxazolone administration. The damage scores were evaluated as described in the “Methods” section. Data are expressed as a column chart. ^*^ *P* < 0.05, ^**^ *P* < 0.01, compared with that of the Blank group. ^#^ *P* < 0.05, ^##^ *P* < 0.01, compared to that of the oxazolone-treated group

Colonic damage scores were significantly higher in OXA-treated mice compared to those of the Blank group mice (*P* < 0.01). Macroscopic analysis indicated that in the OXA-treated group, inflammation was mainly localized in the distal part of the colon, which shortened and thickened, as well as presented signs of colonic edema and hemorrhagic changes (Fig. 2e).

Carrageenan alone showed no impact on the colons of animals, wherein the colonic mucosa was apparently normal, and symptoms of congestion, edema, and erosion were not detected (Fig. 2b–d). No significant differences in colonic macroscopic scores of mice from the Blank group mice and those of the LOW, MED, and HIG treatment groups were observed. Figure 2f–h reveals that OXA-induced colonic damage was markedly augmented in mice pretreated with different concentration of carrageenan. The colons were more reddish due to the production of a bloody stool compared to those of the OXA-treated mice, suggesting that a more severe intestinal inflammation had occurred in the LOW + OXA, MED + OXA, and HIG-OXA-treated mice (Fig. 2f–g). The colonic damage scores of the LOW + OXA, MED + OXA, and HIG + OXA groups were higher than that of the OXA-induced group (*P* < 0.05, Fig. 2i). Among these, the MED + OXA group showed the highest score of 3.75. A 1.55-fold higher colonic damage score was observed in the MED + OXA group compared to the OXA-treated group (*P* < 0.01). These results indicate that carrageenan can synergistically enhance OXA-induced inflammation.

### Effects of κ-carrageenan on the histological changes of OXA-induced colonic damage

**Fig. 3:**
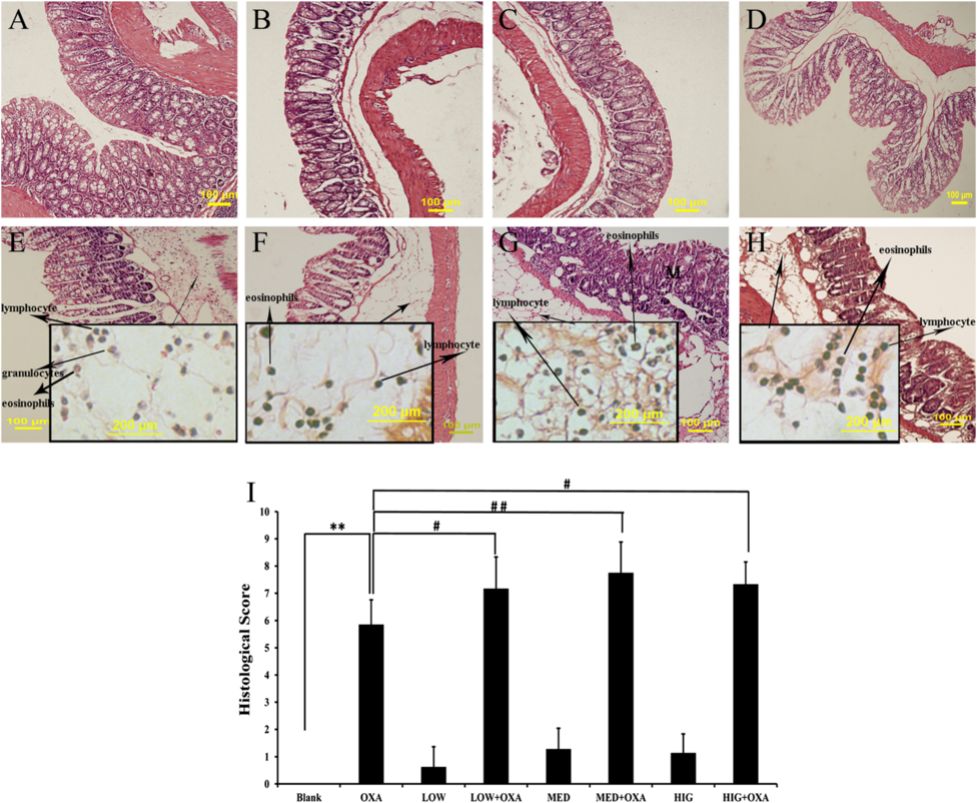
Effect of κ-carrageenan on colonic histological changes and colonic histological scores of oxazolone-treated mice. Representative images of the histological features of the colonic tissue of blank (**a** Blank group; **b** LOW group; **c** MED group; **d** HIG group; **e** OXA group; **f** LOW + OXA group; **g** MED + OXA group; **h** HIG + OXA group. HE: ×200, Higher magnifications of the pictures: ×600). **i** Histological grading of colitis in oxazolone-induced mice. Histological grading was determined as described in the “Methods” section. Data are expressed as a histogram plot. ^**^ *P* < 0.01, compared to that of the Blank group; ^#^ *P* < 0.05, ^##^ *P* < 0.01, compared to that of the oxazolone-treated group

No histological differences in the effect of κ-carrageenan on OXA-induced colonic damage between the carrageenan-treated groups and the Blank group were observed. The colonic tissue of the Blank group showed normal features such as an adequate number of goblet cells and intact crypts (Fig. 3a). Figure 3e shows the colonic sections of OXA-exposed mice with a lower number of goblet cells and epithelial cells, and an increase in lymphocyte infiltration, accompanied by a mixed inflammatory infiltrate of granulocytes, relative to that observed in mice of the Blank group.

Histological analysis showed that carrageenan was effective in aggravating OXA-induced colitis (Fig. 3f–h). The colonic sections from the LOW + OXA, MED + OXA, and HIG + OXA-treated mice revealed mucosal inflammation, which was characterized by lymphocyte cell infiltration, as well as a significant reduction in the number of goblet cells. A 1.22-fold augmentation in histologic score was obtained in the LOW + OXA group (*P* < 0.05), a 1.32-fold increase in the MED + OXA group (*P* < 0.01), and a 1.25-fold increase in the HIG + OXA group relative to the OXA-treated group (*P* < 0.05) (Fig. 3i).

### Analysis of histological changes of the colon by using scanning electron microscopy

**Fig. 4:**
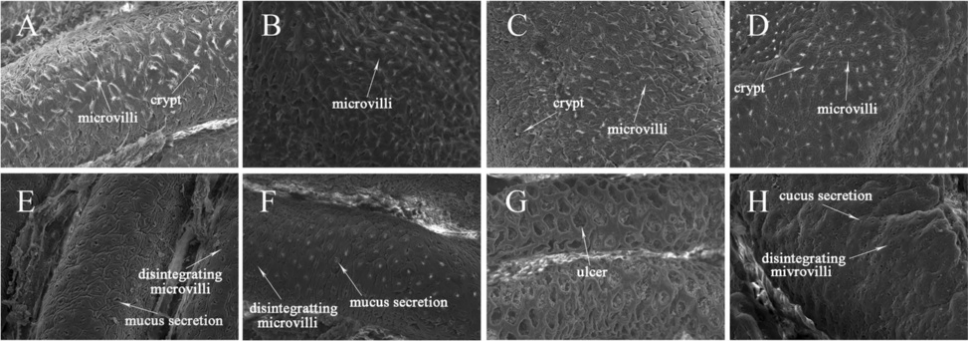
Scanning electron microscopy of histological changes. **a** Blank group; **b** LOW group; **c** MED group; **d** HIG group; **e** OXA group; **f** LOW + OXA group; **g** MED + OXA group; **h** HIG + OXA group. ×200 magnification

Intact microvilli, normal epithelial cell surface, and crypts were observed by scanning electron microscopy in the Blank group (Fig. 4a). No significant differences in the epithelial mucosa of the colon between the LOW, MED, and HIG groups and the Blank group were observed, with all groups showing normal features (Fig. 4b–d). However, at higher magnification, damage to the epithelial mucosa surface and disintegration of the microvilli were observed in the colon of the OXA-treated group (Fig. 4e). Figure 4 shows that carrageenan was effective in aggravating OXA-induced colonic damage. The histological changes of the colon were markedly exacerbated in the LOW + OXA, MED + OXA, and HIG + OXA groups (Fig. 4f–h). Scanning electron microscopy showed more pronounced damage to the epithelial cell surface, disintegration of the microvilli, excessive mucus secretion at crypt openings, extensive distribution of ulcers on the colonic surface were observed in the tissues of mice belonging to the MED + OXA group (Fig. 4e).

### κ-Carrageenan administration promoted the OXA-induced decrease in the ratio of CD4^+^CD25^+^ regulatory T cells (Tregs)

**Fig. 5:**
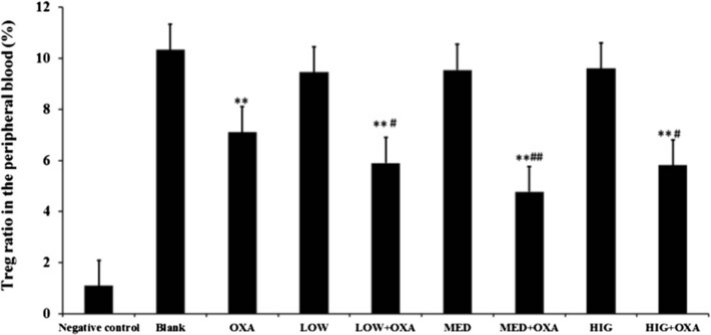
Ratio of CD4 + CD25+/CD4+ regulatory T cells (Tregs) in the peripheral blood of mice. ^**^ *P* < 0.01, compared to that of the Blank group. ^#^ *P* < 0.05, compared to that of the oxazolone-treated group

Treg cells regulate responses of the immune system. Figure 5 reveals that the ratio of CD4^+^CD25^+^/CD4^+^ in the peripheral blood of OXA-treated group was significantly lower than that observed in the Blank group (*P* < 0.01). No apparent effects of different concentrations of κ-carrageenan alone on the proportion of Treg cells were observed. On the other hand, κ-carrageenan aggravated the decrease in CD4^+^CD25^+^/CD4^+^ cells in the peripheral blood of OXA-treated mice. A 1.206-fold reduction in CD4^+^CD25^+^/CD4^+^ was observed in the LOW + OXA group (*P* < 0.05) and 1.22-fold decrease in the HIG + OXA group compared to that of the OXA-treated group (*P* < 0.05). The CD4^+^CD25^+^/CD4^+^ ratio of the MED + OXA group showed 1.49-fold decrease compared to that of the oxazolone group (*P* < 0.01). These findings demonstrate that OXA causes an immune imbalance in mice, whereas carrageenan augments this imbalance.

### κ-Carrageenan promotes cytokine production in OXA-induced mice colonic inflammation

The levels of seven cytokines in the serum were determined by flow cytometry by using the Mouse Inflammation Cytometric Bead Array (CBA) assay. Of these cytokines, TNF-α and IL-6 as classified as pro-inflammatory cytokines; IFN-γ and IL-2 are Th1 cytokines; IL-4, IL-10 are Th2 cytokines; and IL-17A is a Th17 cytokine.

Interestingly, administration of carrageenan alone resulted in the upregulation of the seven cytokines, except for IL-17A, but no statistical difference (*P* > 0.05, Table 2). However, OXA treatment induced a significant elevation in the secretion of TNF, IL-6, and IL-10 (*P* < 0.01). Mice pretreated with medium-dose (8.3 mg/kg) of carrageenan showed a significant increase in the OXA-induced production of IL-10, IL-6, and TNF (*P* < 0.01). Of the seven cytokines, IL-6 showed the highest increase (3.82-fold) compared to that of the OXA group (Table 2).

**Table 2:** The levels of the inflammatory cytokines IL-10, IL-17A, TNF, IFN-γ, IL-6, IL-4, and IL-2 in mouse serum (pg/ml, mean ± SEM)

Group	IL-10	IL-17A	TNF	IFN-γ	IL-6	IL-4	IL-2
Blank	1.73 ± 0.15	2.31 ± 0.35	3.71 ± 0.20	1.87 ± 0.08	2.61 ± 0.15	1.79 ± 0.11	1.80 ± 0.32
OXA	6.48 ± 1.19 ^**^	2.94 ± 0.99	10.98 ± 0.78^**^	1.81 ± 0.38	47.6 ± 2.64 ^**^	2.34 ± 0.43	2.43 ± 0.09
LOW	3.42 ± 0.45	2.91 ± 1.00	6.18 ± 0.70	2.64 ± 0.44	3.57 ± 0.50	2.33 ± 0.33	2.14 ± 0.28
LOW + OXA	11.34 ± 2.4^**#^	3.07 ± 0.83	26.62 ± 3.07^**##^	1.69 ± 0.27	92.32 ± 3.36^**#^	2.70 ± 0.27^*^	2.28 ± 0.27
MED	2.27 ± 0.78	2.34 ± 2.08	6.40 ± 0.35	2.58 ± 0.91	4.31 ± 0.55	2.20 ± 0.60	2.08 ± 0.21
MED + OXA	19.67 ± 2.35^**##^	2.56 ± 0.44	35.0 ± 3.13^**##^	1.88 ± 0.21	182.17 ± 7.85^**##^	2.49 ± 0.12^**^	2.57 ± 0.18
HIG	2.80 ± 0.74	2.07 ± 0.17	5.95 ± 0.75	1.90 ± 0.14	3.64 ± 0.39	2.36 ± 0.68	2.60 ± 0.24
HIG + OXA	12.32 ± 2.57^**#^	2.56 ± 0.34	30.63 ± 2.83^**##^	1.61 ± 0.08	107.17 ± 3.74^**#^	2.66 ± 0.11^**^	2.16 ± 0.45

Notes: ^*^ *P* < 0.05, compared to that of the blank group. ^**^ *P* < 0.01, compared to that of the blank group. ^#^ *P* < 0.05, compared to that of the oxazolone-treated group. ^##^ *P* < 0.01, compared to that of the TNBS-treated group

### The expression levels of TLR4, NF-κB, PCNA, and FOXp3 mRNA in the colonic mucosa of experimental mice

**Fig. 6:**
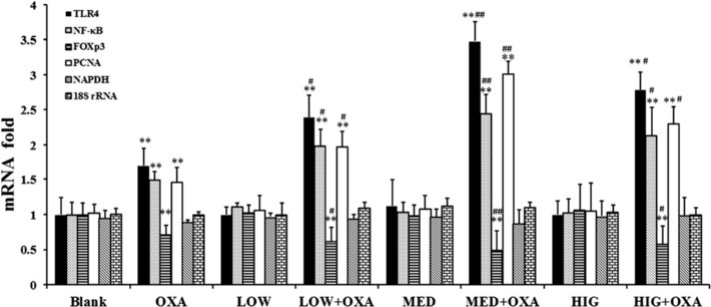
The impact of κ-carrageenan on the expression of inflammatory response-related proteins in the colonic mucosa of experimental mice. 200× magnification

No differences in the levels of the four proteins were observed between Blank group and κ-carrageenan alone-treatment group. However, OXA exposure and induction of inflammation was associated with a significant increase in the level of mRNA expression of TLR4, NF-κB, and PCNA in mouse colonic tissues. Carrageenan pretreatment resulted in a significant increase in the level of mRNA expression of TLR4, NF-κB, and PCNA compared to that of the OXA control group. However, a decrease in the level of FOXp3 mRNA expression was observed after OXA treatment (*P* < 0.01), and pretreatment with different doses of κ-carrageenan with OXA resulted in a significantly synergistic decreased in the FOXp3 mRNA expression compared to that of the OXA group (Fig. 6; *P* < 0.05).

### Synergistic effects of κ-carrageenan on OXA-induced protein expression in colonic mucosa of experimental mice

**Fig. 7:**
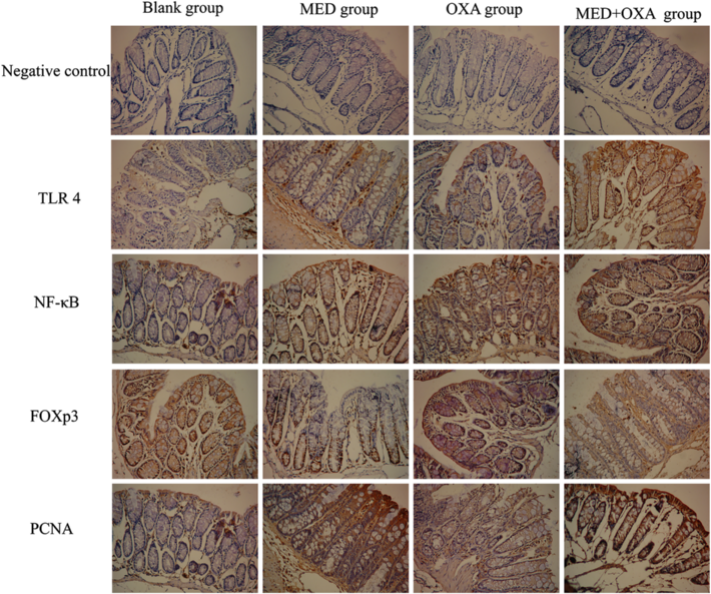
Effect of κ-carrageenan on the level of mRNA expression of TLR4, NF-κB, FOXp3, and PCNA. Real-time quantitative RT-PCR was used to measure the level of mRNA expression of TLR4, NF-κB, FOXp3, and PCNA. ^*^ *P* < 0.05, ^**^ *P* < 0.01 relative to that of the Blank group. ^#^ *P* < 0.05, ^##^ *P* < 0.01 compared to that of the OXA-treated group

The impact of carrageenan on the expression of TLR4, NF-κB, and PCNA-protein levels in the colonic mucosa of experimental mice was immunohistochemically analyzed. Table 2 shows that κ-carrageenan alone did not apparently affect the expression of TLR4, NF-κB, and PCNA in colonic epithelial cells. The expression levels of TLR4, NF-κB, and PCNA in the colonic epithelium increased after OXA injection (*P* < 0.01). Mice pretreated with different doses of carrageenan showed a significant increase in the OXA-induced expression of TLR4, NF-κB, and PCNA (*P* < 0.05). The most significant increase in the expression levels of these proteins was observed in the MED + OXA group. A 1.51-fold increase in TLR4 expression in colonic mucosa was detected in the MED + OXA group (*P* < 0.01), whereas a 1.14-fold upregulation in NF-κB expression in colonic mucosa was observed in the MED + OXA group (*P* < 0.01) and a 1.79-fold increase in PCNA expression in the MED + OXA group compared to that of the OXA-treated group was detected (*P* < 0.01) (Fig. 7, Table 3).

**Table 3:** Impact of carrageenan on the expression of various proteins in mouse colonic mucosa (Au, mean ± SEM)

Group	Negative control	TLR4	NF-κB	FOXp3	PCNA
Blank	1.57 ± 0.48	6.24 ± 0.48	7.01 ± 0.73	25.97 ± 0.38	5.30 ± 0.24
OXA	1.84 ± 0.31	13.83 ± 0.21^**^	18.07 ± 0.82^**^	16.51 ± 0.72^**^	13.54 ± 0.43^**^
LOW	2.65 ± 0.19	6.45 ± 0.32	7.8 ± 1.22	24.23 ± 0.58	5.86 ± 0.20
LOW + OXA	2.74 ± 0.23	15.25 ± 0.23^**#^	20.26 ± 1.40^**#^	14.24 ± 0.65^**#^	15.96 ± 0.69^**#^
MED	2.53 ± 0.28	6.63 ± 0.25	7.95 ± 1.26	24.50 ± 0.75	6.19 ± 0.18
MED + OXA	2.36 ± 0.29	17.61 ± 0.45^**##^	26.68 ± 0.83^**##^	6.48 ± 0.68^**##^	24.41 ± 0.27^**##^
HIG	2.59 ± 0.23	6.56 ± 0.20	7.33 ± 1.16	24.56 ± 0.75	5.99 ± 0.28
HIG + OXA	77.6 ± 15.3	15.07 ± 0.78^**#^	21.33 ± 1.90^**#^	14.18 ± 0.86^**#^	16.98 ± 1.70^**##^

Notes: **P < 0.01, compared to that of the Blank group; ^#^ *P* < 0.05, ^##^ *P* < 0.01, compared to that in the oxazolone-treated group

Table 3 shows that the expression levels of Foxp3 expression in the colonic epithelium decreased after OXA injection (*P* < 0.01), whereas carrageenan treatment significantly decreased the level of OXA-induced expression of FOXp3 (Fig. 6; Table 3; *P* < 0.05).

### κ-Carrageenan enhances oxazolone-induced activation of TLR4 and NF-κB in experimental mice

**Fig. 8:**
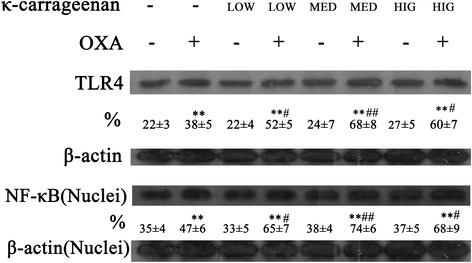
κ-Carrageenan enhanced oxazolone-induced activation of NF-κB. The protein levels of TLR4 and NF-κB p65 were analyzed by western blotting. The data are presented as the density ratio vs. β-actin. **P < 0.01, compared to that of the Blank group. # *P* < 0.05, ## *P* < 0.01, compared to that of the oxazolone-treated group

NF-κB is one of the most important transcription factors that regulate the expression of numerous pro-inflammatory cytokines. We examined whether κ-CGN enhances oxazolone-induced NF-κB activation. Figure 8 shows that treatment with κ-carrageenan alone resulted in a slight increase in oxazolone-induced TLR4 and nuclear levels of the p65 subunit of NF-κB. However, oxazolone significantly increased TLR4 and NF-κB levels (*P* < 0.01). In addition, pretreatment with different doses of κ-carrageenan markedly promoted the expression of TLR4 and NF-κB in oxazolone-stimulated mice. A 1.78-fold increase in TLR4 expression in colonic tissues was detected in the MED + OXA group (*P* < 0.01) and a 1.57-fold upregulation in NF-κB expression in the MED + OXA group compared to that of the OXA-treated group (*P* < 0.01) (Fig. 8, Table 3).

## Discussion

Although the FDA and JECFA consider carrageenan as a safe saccharide [1, 5, 11], studies have shown that it induces ulcerations in animal models, suggests that it poses a carcinogenic risk to humans [1–3, 27]. This finding has sparked controversy and intense debate, and the mechanism underlying this pathological condition has remained uncertain. The present study presents a possible mechanism of carrageenan that involves an inflammatory response.

BALB/c is a mouse strain that is susceptible to OXA-induced inflammation [24, 28]. In this research, we used OXA to establish an acute gut inflammation BALB/c mouse model, which resembles ulcerative colitis in pathology and the immune response inof human. It has been reported that the initial toxic effects of OXA leads to a flooding of the lamina propria and inducing an increase in the secretion of Th2 cytokines IL-4 and IL-5, which in turn leads to further inflammation. [24, 29]. Moreover, rectal instillation of 150 μL of 1 % OXA is enough to lead to an accelerated inflammation lasting for 1–2 days by a Th2 response in the distal half of the mouse colon of the mice, thereby resulting in either rapid recovery or death [24]. Therefore, one day after injection of OXA was decided as the experimental time to detect the aggravating effect of carrageenan on the inflammatory response.

The average daily intake of carrageenan in the infant formula is 0.3 g/L [2], amounting to 240 mg/5.8 kg/day (≈41.7 mg/kg/day). In the typical Western diet, the daily intake of carrageenan is considerably more than 500 mg/60 kg/day (≈8.3 mg/kg/day) [30]. Bhattacharyya et al. reported that a dose of 50 μg/30 g/day (≈1.7 mg/kg/day) carrageenan induces colonic inflammation in IL-10-deficient mice [18]. Therefore, in the present study, we used these concentrations to investigate the effect of carrageenan on OXA-induced inflammation.

Here, we first discovered that κ-carrageenan aggravates OXA-induced inflammation in BALB/c mice by significantly increasing weight loss, lethality rate, and degree of colonic injury. Of particular interest is our finding that mice pretreated with a medial-dose (8.3 mg/kg) of κ-carrageenan for 14 days resulted in a 37.5 % of mortality rate, whereas that of OXA-treated control mice was 12.5 % (Fig. 2). Histological and microscopic evaluation also confirmed that intragastric administration of κ-carrageenan aggravated OXA-induced inflammation in BABL/c mice. Of the three doses of κ-carrageenan used in the present study, the medium dose showed highest score histological scores. The survival ratio of the OXA + MED group was also the lowest. It is possible that the high concentration of carrageenan induces its formation into a gel in the gut of mice, which in turn reduces its contact without other gut content. In addition, Weiner et al. also observed that intragastric administration of a high dose of food-grade carrageen an just directly affected theresulted in the production of soft stools or development of diarrhea, which are common effects of non-digestible high-molecular weight compounds [31]. Therefore, the aggravated effects of carrageenan do not appear to respond to a traditional dose–response curve.

OXA-induced inflammation is often employed in research investigations on the contribution of the Th2-dependent immune response to intestinal inflammation [24]. The present study showed that OXA-induced inflammation is accompanied by the release of various inflammatory cytokines, not only Th2 type cytokines IL-10, but also pro-inflammatory cytokines, TNF-α and IL-6 [24, 28]. Previous reports have revealed that OXA-induced inflammation is characterized by an upregulation of IL-4 [29]; however, in the present study, although an increased level of IL-4 expression was observed in the OXA group, this was not statistically significant. No increase in the levels of Th1 cytokines IFN-γ, IL-2, and IL-17 was observed with OXA and κ-carrageenan treatment. The synergistic effect of κ-carrageenan on OXA-induced changes is also reflected in the secretion levels of inflammatory cytokines. The secretion of pro-inflammatory cytokines TNF-α and IL-6 in OXA-treated mice were all significantly augmented by κ-carrageenan pretreatment. These findings suggest that carrageenan aggravated the OXA-induced inflammation of intestinal tissues.

CD4^+^CD25^+^ regulatory T cells (Tregs) can regulate and suppress the immune response, thus; therefore, these play an important role in the maintenance of peripheral immune tolerance. The decrease in the number of Tregs often results in a development of various kinds of autoimmune disease such as inflammatory bowel disease (IBD) [32]. The present study showed that the number of Tregs in the peripheral blood of OXA-treated group was lower than that of the Blank group, which further decreased after κ-carrageenan treatment, demonstrating that OXA can cause an imbalance in the immune system of mice, and carrageenan augments this imbalance.

FOXp3 is a transcription factor that plays a major role in the development and maintenance of the immunosuppressive function of Tregs [33]. Galitovskiy et al. observed that the expression of FOXp3 is decreased in the inflamed areas of ulcerative colitis compared to that of the normal colon [34]. This finding is consistentin agreement with the observations of the present study, wherein the intragastric administration of κ-carrageenan significantly decreased the OXA-induced mRNA expression of FOXp3 in the colonic mucosa, indicating that the suppressor activity of FOXp3 might have been abrogated in vivo or was insufficient to counterbalance the acute mucosal inflammation in OXA-treated mice.

Mice pretreated with different doses of carrageenan showed a significant increase in the levels of OXA-induced expression of NF-κB and TLR4. Some studies have demonstrated that carrageenan upregulated interleukin-8 (IL-8) secretion through TLR4, and activated NF-κB in human colonic epithelial cells [16, 20]. Benard et al. also observed that κ-carrageenan induced TNF production via NF-κB activation [21]. Recently, we have beenare exploring the mechanism of underlying the synergistic effect of κ-carrageenan on lipopolysaccharide (LPS)-induced interleukin-8 (IL-8) secretion in HT-29 cells. We found that κ-carrageenan pretreatment increased LPS-stimulated IL-8 secretion. The TLR4-NF-κB pathway was activated, as evidenced by the enhancement of Bcl10 (B-cell CLL/lymphoma 10) expression, IκBα phosphorylation, and nuclear NF-κB localization (Wu, wuweixiehou@163.com). These findings demonstrate that intragastric administration with κ-carrageenan significantly activated intestinal inflammation by upregulating TLR4 and NF-κB in colonic mucosa.

## Conclusion

In conclusion, intrarectal injection of OXA in mice causes colonic damage and inflammation. Treatment of mice with κ-carrageenan significantly aggravated OXA-induced inflammation in BALB/c mice. The results of the present study suggest that the synergistic effect of κ-carrageenan might be linked to the induction of an imbalance in the expression of intestinal inflammatory cytokines, which then magnified the existing intestinal inflammation. Therefore, under the intestinal “unhealthy” state, carrageenans may serve as potential inflammatory agents that impart certain harmful effects.
